# Astragaloside II Ameliorated Podocyte Injury and Mitochondrial Dysfunction in Streptozotocin-Induced Diabetic Rats

**DOI:** 10.3389/fphar.2021.638422

**Published:** 2021-03-16

**Authors:** Jun Su, Chongting Gao, Ling Xie, Ying Fan, Yilan Shen, Qunwei Huang, Niansong Wang, Youhua Xu, Nizhi Yang, Dingkun Gui

**Affiliations:** ^1^ Department of Nephrology, Shanghai Jiao Tong University Affiliated Sixth People’s Hospital, Shanghai, China; ^2^ Shanghai Ocean University, Shanghai, China; ^3^ Faculty of Chinese Medicine, State Key Laboratory of Quality Research in Chinese Medicine, Macau University of Science and Technology, Macao, China; ^4^ Department of Nephrology, Guangdong Provincial Hospital of Chinese Medicine, Guangzhou, China

**Keywords:** diabetic nephrology, astragaloside II, podocyte injury, mitochondrial dynamics, mitophagy

## Abstract

Astragaloside II (AS II), a novel saponin purified from Astragalus membranes, has been reported to modulate the immune response, repair tissue injury, and prevent inflammatory response. However, the protective effects of AS II on podocyte injury in diabetic nephropathy (DN) have not been investigated yet. In this study, we aimed to investigate the beneficial effects of AS II on podocyte injury and mitochondrial dysfunction in DN. Diabetes was induced with streptozotocin (STZ) by intraperitoneal injection at 55 mg/kg in rats. Diabetic rats were randomly divided into four groups, namely, diabetic rats and diabetic rats treated with losartan (10 mg·kg^−1^·d^−1^) or AS II (3.2 and 6.4 mg·kg^−1^·d^−1^) for 9 weeks. Normal Sprague-Dawley rats were chosen as nondiabetic control group. Urinary albumin/creatinine ratio (ACR), biochemical parameters, renal histopathology and podocyte apoptosis, and morphological changes were evaluated. Expressions of mitochondrial dynamics-related and autophagy-related proteins, such as Mfn2, Fis1, P62, and LC3, as well as Nrf2, Keap1, PINK1, and Parkin, were examined by immunohistochemistry, western blot, and real-time PCR, respectively. Our results indicated that AS II ameliorated albuminuria, renal histopathology, and podocyte foot process effacement and podocyte apoptosis in diabetic rats. AS II also partially restored the renal expression of mitochondrial dynamics-related and autophagy-related proteins, including Mfn2, Fis1, P62, and LC3. AS II also increased the expression of PINK1 and Parkin associated with mitophagy in diabetic rats. Moreover, AS II facilitated antioxidative stress ability via increasing Nrf2 expression and decreasing Keap1 protein level. These results suggested that AS II ameliorated podocyte injury and mitochondrial dysfunction in diabetic rats partly through regulation of Nrf2 and PINK1 pathway. These important findings might provide an innovative therapeutic strategy for the treatment of DN.

## Introduction

Diabetic nephrology (DN) is one of the most common causes of end-stage renal disease (ESRD) (Lindblom et al., 2015; Papadopoulou-Marketou et al., 2017). Podocytes are highly specialized, terminal differentiated cells that play essential roles in maintaining glomerular filtration barrier integrity (Liu et al., 2017). Podocyte apoptosis played critical roles in the progression of DN (Susztak et al., 2006). In the hyperglycemic state, podocytes undergo cytoskeletal rearrangement, increased apoptosis, and abnormal autophagy, which are manifested as foot process effacement and podocyte loss (Herman-Edelstein et al., 2011). Mitochondria are dynamic organelles that undergo dynamic cyclic balance of fusion and fission, modulated by profission proteins (Fis1 and Drp1) and profusion proteins (OPA1 and Mfn1/2) (Zhan et al., 2015; Xiao et al., 2017). Mitochondrial dysfunction is strictly essential for the development of diabetes and its complications (Bhargava and Schnellmann, 2017). Podocytes are sensitive to mitochondrial impairment (Zhou et al., 2019). Evidence was obtained from excessive mitochondrial fragmentation in podocytes of the STZ-induced mice model (Wang et al., 2012). Excessive mitochondrial fission is a key mediator that produces a massive amount of reactive oxygen species (ROS) and apoptogenic proteins (e.g., caspase-3), eventually activating the death pathway of mitochondria (Xiao et al., 2017). These studies suggest that the perturbation of mitochondrial dynamics is likely associated with renal function deterioration and podocyte injury.

Moreover, increased mitochondrial fragments caused by mitochondrial damage promote autophagic clearance of mitochondria (Youle and van der Bliek, 2012). Dysfunctional mitochondria are specially targeted for degradation via PTEN-induced putative kinase1 (PINK1)/Parkin pathway (Higgins and Coughlan, 2014). An average reduction of autophagic vacuoles volume densities in diabetic rats was observed (Han et al., 1992). Inhibition of autophagy can be reversed by insulin replacement in the kidneys of diabetic rats, suggesting that glucose has a certain degree of influence on cellular autophagy (Han et al., 1992). Mitophagy inhibition also occurred in damaged diabetic podocytes, which thereby developed marked glomerulosclerosis and albuminuria (Zhou et al., 2019). Therefore, the damage of autophagy/mitophagy is one of the most important factors leading to podocyte injury in DN.

Furthermore, there was a potential relationship between P62 and Kelch-like ECH-associated protein 1 (Keap1)/nuclear factor erythroid-derived-2-like 2 (Nrf2) pathway that associated with oxidative stress (Jiang et al., 2015). Nrf2 is a key regulator of cellular antioxidant response and binds to the adaptor protein Keap1, which is inactive under physiological conditions (Sun et al., 2016). However, large amounts of intracellular P62 are accumulated when autophagy is suppressed, causing the conformational change of Keap1, resulting in increased release of Nrf2, thereby initiating downstream gene expression, including PINK1 protein associated with mitophagy (Sun et al., 2016; Lin et al., 2018). Thus, protecting podocyte injury by alleviating mitophagy may be an effective therapeutic strategy for treating DN.

Diabetes or diabetic nephrology is considered as “consumptive thirst (Xiaoke)” in Traditional Chinese medicine (TCM) ancient literature. The relevant disease name, symptoms, complications, and treatment of consumptive thirst were recorded in Huangdi’s Internal Classic, the foundation of basic Chinese medical theories (Luo and Wang, 2018). TCM has a long history in the treatment of diabetic complications in China. Increasing evidence demonstrated that the Chinese herbs “benefiting vital energy and activating blood circulation (Yiqi Huoxue)” ameliorated proteinuria in patients with diabetic nephrology (Fan et al., 2009). *Astragalus membranaceus* (huang qi) is one of the most widely used ‘‘benefiting vital energy (Yiqi)” herbs in TCM to treat various diseases. *Astragalus membranaceus* was found to have renal protective effects on DN (Li et al., 2011). It has been reported that Astragaloside II (AS II), one of the active constituents of Astragalus membranaceus, exerted anti-inflammatory and wound-healing effects in inflammatory bowel disease (Lee et al., 2017; Qiao et al., 2019). AS II also reversed P-glycoprotein-mediated multidrug resistance of human hepatic cancer cells (Huang et al., 2012) and induced osteogenic activities of osteoblasts (Kong et al., 2012). However, the beneficial effects of AS II on podocyte injury in DN have not been investigated yet. In the present study, we aimed to study the effects of AS II on podocyte injury and mitochondrial dysfunction in DN and then provide a novel strategy for treating DN.

## Materials and Methods

### Drug Preparation

AS II (with a purity of over 98%) was purchased from Shanghai Jingke Chemical Technology Co., Ltd. (Shanghai, China), and was suspended in olive oil (Osaka, Japan), as a vehicle for administration to rats. Losartan was purchased from Merck Sharp & Dohme Limited (Merck Sharp & Dohme, Australia) and suspended in 0.5% methylcellulose solution. Streptozotocin (STZ) was purchased from Sigma-Aldrich Company (Sigma-Aldrich, United States) and was dissolved in the citrate buffer (0.1 M, pH 4.5) (Chen et al., 2014).

### Animals and Treatment

All the animal experiments were carried out according to the Guide for the Care and Use of Laboratory Animals proposed by National Institutes of Health (NIH) and were approved by the Animal Ethics Committee of Shanghai Jiao Tong University Affiliated Sixth People’s Hospital. Healthy 8-week-old male Sprague-Dawley rats weighing 200–250 g were housed under a constant 12 h light–dark cycle at a temperature between 21 and 23°C and allowed free access to food and water. Diabetes was induced in rats by a single intraperitoneal injection of STZ at 55 mg/kg, according to our previous study (Xie et al., 2020). Seventy-two hours after intraperitoneal injection of STZ, the blood glucose level was measured from the tail vein, and rats with a blood glucose level above 16.7 mmol/L were considered as diabetic rats (Xie et al., 2020). The diabetic rats were then randomly divided into four groups (*n* = 7/each group): 1) STZ-induced diabetic rats (STZ), received an equal volume of olive oil; 2) diabetic rats treated with losartan at 10 mg·kg^−1^·d^−1^; 3) diabetic rats treated with low-concentration of AS II at 3.2 mg·kg^−1^·d^−1^ (ASIIL); 4) diabetic treated with high-concentration of AS II at 6.4 mg·kg^−1^·d^−1^ (ASIIH). Normal rats were chosen as the normal control (Con, nondiabetic rats). All treatment interventions were administrated at two weeks after STZ injection and were administered via oral gavage to rats for nine weeks. Rats were kept in individual metabolic cages for 24 h urine collections at the end of 0 and 9 weeks of treatments. Rats were then anesthetized with pentobarbital sodium and the blood samples were collected through the abdominal aorta for measuring biochemical parameters by using an automatic biochemistry analyzer, and the kidneys were harvested immediately.

### Urine and Blood Biochemical Characteristics

Urine was centrifuged at 3,500 rpm for 10 min at 4°C and the supernatants of urine were measured for the urinary albumin and urinary creatinine by an automatic biochemistry analyzer (Hitachi Model 7600-120E, Japan). Urinary albumin excretion was expressed as urinary albumin/creatinine ratio (ACR). Blood was centrifuged at 3,500 rpm for 10 min at 4°C and the supernatants of blood were measured for blood glucose (GLU) by an automatic biochemistry analyzer (Hitachi Model 7600-120E, Japan).

### Renal Histology

The kidneys were fixed in 10% buffered formalin and embedded in paraffin, cut into 4 μm sections, and stained with hematoxylin and eosin (HE) and periodic acid-Schiff (PAS). The protocol of HE and PAS staining was described previously (Zhai et al., 2019). Sections were dried for 30 min at 65°C and then were deparaffinized and rehydrated through dimethylbenzene (I), dimethylbenzene (II), 100% ethanol (I), 100% ethanol (II), 95% ethanol, 90% ethanol, 80% ethanol, and deionized water, 10 min for each step. Subsequently, kidney sections were stained by HE and PAS and detected by light microscopy (Leica, Germany). To assess and calculate the injury of renal histology in HE and PAS staining, 20 cortical fields (×400 magnification) were chosen randomly by two blinded investigators. The sections from each renal tissue were graded and scored for the mesangial matrix index according to the following scale: Score 0, no lesion; Score 1+, <25% of the glomerulus sclerosis; Score 2+, 25–50% of the glomerulus sclerosis; Score 3+, 50–75% of the glomerulus sclerosis; Score 4+, >75% of the glomerulus sclerosis (Fujihara et al., 2000).

### Transmission Electron Microscopy Studies

Morphological and structural characteristics of podocyte were examined by transmission electron microscopy (TEM). Renal cortices were cut into 1 mm^3^ pieces on ice and immediately fixed in 2.5% glutaraldehyde for 2 h at 4°C and washed twice in the same buffer. After dehydration with different concentrations of ethyl alcohol, renal cortices were embedded in epoxy resin 48 h at 60°C. Ultrathin sections were stained with uranyl acetate and lead citrate and examined by TEM. The number of foot processes (FP) in kidney sections was counted by a blinded observer as previously described (Chen et al., 2014).

### Immunohistochemistry

Paraffin-embedded kidney tissues were deparaffinized and rehydrated, xylene, ethanol, and quenched by 3% H_2_O_2_ for 15 min. The sections were blocked by 10% goat serum for 1 h at room temperature. Then, the sections were incubated overnight at 4°C with the following primary antibodies: WT1 (1:200, ABclonal, China), nephrin (1:1,000, Abcam, United States), cleaved-caspase-3 (1:200, Abcam, United States), p62 (1:50, Proteintech, China), LC3 (1:600, Proteintech, China), Mfn2 (1:2000, Proteintech, China), Fis1 (1:100, Abcam, United States), PINK1 (1:200, Proteintech, China), parkin (1:1,000 Proteintech, China), Nrf2 (1:200, Abcam, United States), and Keap1 (1:1,500, Servicebio, China). The secondary antibodies (Dako, United States) were added to the sections, which were incubated for 1 h at 37°C. Finally, the sections were counterstained using diaminobenzidine and hematoxylin staining and then were rinsed after dehydration. Images were then collected using a light microscope (Leica, Germany). The positive-staining cells in each kidney section were semiquantified by ImageJ software (NIH, Bethesda, MD, United States).

### Immunofluorescence Staining and TUNEL Assay

Triple immunofluorescence labeling, including terminal deoxynucleotidyl transferase-mediated dUTP nick-end labeling (TUNEL) assay, WT1, and 4,6-diamidino-2-phenylindole (DAPI), was used to examine the podocyte apoptosis. TUNEL assay was conducted on frozen sections of kidney samples (4 μm) according to the manufacturer’s instructions (*In Situ* Apoptosis Detection Kit, Merck, S7165). Afterward, the sections were incubated with the primary antibody of WT1 diluted in PBS (1:100, ABclonal, China) at 4°C overnight. Then, secondary antibodies conjugated to Alexa Fluor 488 (abcam, United States) were added to the sections for 1–2 h and counterstained with DAPI. The triple-positive cells (WT1, TUNEL, and DAPI) were identified as the apoptotic podocytes. All fluorescent images were visualized under a confocal microscope (Olympus, Japan) by two blinded investigators.

### Western Blotting Assay

Kidney tissues were homogenized in RIPA lysis buffer containing protease and phosphatase inhibitor cocktail on ice with a homogenizer. The supernatants were collected after centrifuging at 12,000 rpm for 24 min at 4°C. Protein concentration of the supernatants was calculated by the bicinchoninic acid (BCA) kit assay (Biosharp, China). The tissue lysates, mixed with an equal amount of 5× SDS loading buffer, were denatured in boiling water for 10 min. Samples were separated by SDS-polyacrylamide gel electrophoresis (SDS-PAGE) and were transferred onto polyvinylidene difluoride (PVDF) membranes, which were then blocked with Tris-buffered saline Tween-20 (TBST) containing 5% nonfat milk for 1 h at RT. Next, membranes were incubated overnight at 4°C with the following primary antibodies: nephrin (1:1,000, Abcam, United States), cleaved-caspase-3 (1:1,000, Abcam, United States), p62 (1:1,000, Abcam, United States), LC3 (1:1,000, Abcam, United States), Mfn2 (1:1,000, Cell Signaling Technology, United States), Fis1 (1:1,000, Abcam, United States), PINK1 (1:200, Abcam, United States), parkin (1:1,000, Abcam, United States), Nrf2 (1:1,000, Abcam, United States), Keap1 (1:1,000, Abcam, United States), and β-actin (1:5,000, Abcam, United States). After washing for 5–10 min three times with TBST, membranes were incubated with HRP-bounded secondary antibodies (Abcam, United States) for 1 h at RT and washed 5–10 min for three times with TBST again. Finally, proteins were visualized by ECL and ImageJ was used to determine band intensity. Each blot was independently repeated three times.

### Real-Time Quantitative PCR Analysis

Total RNA was extracted from kidney tissues using TRIzol (Invitrogen, Carlsbad, CA) and reverse-transcribed to cDNA with a Reverse Transcription Kit (Takara, Japan) following the manufacturer’s protocol. Real-time PCR was performed by Light Cycler 480 system (Roche, United States) using SYBR Green Master Mix (Vazyme, United States). The primer sequences are as follows: nephrin: 5′-CGT​GCT​GGT​GAT​GAC​TGT​ACG (forward) and 5′-CGT​TCT​TGT​TCT​CCG​ATT​GTG (reverse); cleaved-caspase-3: 5′- CTC​GGT​CTG​GTA​CAG​ATG​TCG (forward) and 5′-TGG​CTC​AGA​AGC​ACA​CAA​AC (reverse); Mfn2: 5′-ACC​ATC​AGT​AGC​CAA​TCT​GGA​C (forward) and 5′-AGA​GCA​GGG​ACA​TCT​CGT​TTC (reverse); Fis1: 5′-CTG​GAC​TCA​TTG​GAC​TGG​CTG​TG (forward) and 5′-AGG​AAG​GCG​GTG​GTG​AGG​ATG (reverse). The target gene expression was calculated using the 2^−ΔΔct^ method and normalized to β-actin.

### Statistical Analysis

Data were analyzed using GraphPad Prism software (version 7.0) and presented as means ± standard deviation (SD). Differences between two groups were compared by unpaired Student's *t*-tests and differences among multiple groups were performed using one-way analysis of variance (ANOVA). Data were considered statistically significant if *p* < 0.05.

## Results

### Effects of AS II on Serum and Urine Levels of Biochemical Characteristics in Diabetic Rats

**FIGURE 1:**
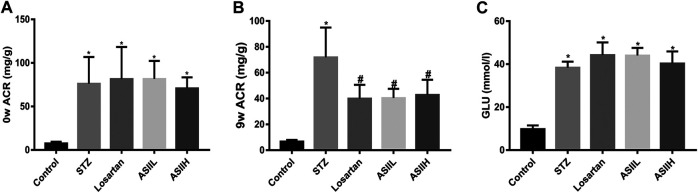
Effects of AS II on serum and urine levels of biochemical characteristics in diabetic rats. **(A)** ACR levels of each group before medicine intervention. **(B)** ACR levels after 9 weeks of treatment. **(C)** The changes of blood glucose (GLU) levels after treatment for 9 weeks. All data were expressed as mean ± SD (*n* = 7). ∗*p* < 0.05 vs. control group; *p* < 0.05 vs. STZ group.

To identify the renoprotective effects of AS II in STZ-induced diabetic rats, the levels of blood glucose (GLU) and urinary ACR were examined. Compared with the normal control rats, ACR level at baseline was markedly increased in diabetic rats (Figure 1A). However, treatment with AS II or losartan significantly reduced the level of ACR at the end of 9 weeks after STZ injection (Figure 1B). These data suggested that AS II treatment attenuated albuminuria in STZ-induced diabetic rats. However, no significant difference in the level of GLU was observed between AS II and losartan treatment groups, which indicated that AS II had no direct effect on the levels of blood glucose (Figure 1C).

### Effects of AS II on Renal Histopathology and Podocyte Injury

**FIGURE 2:**
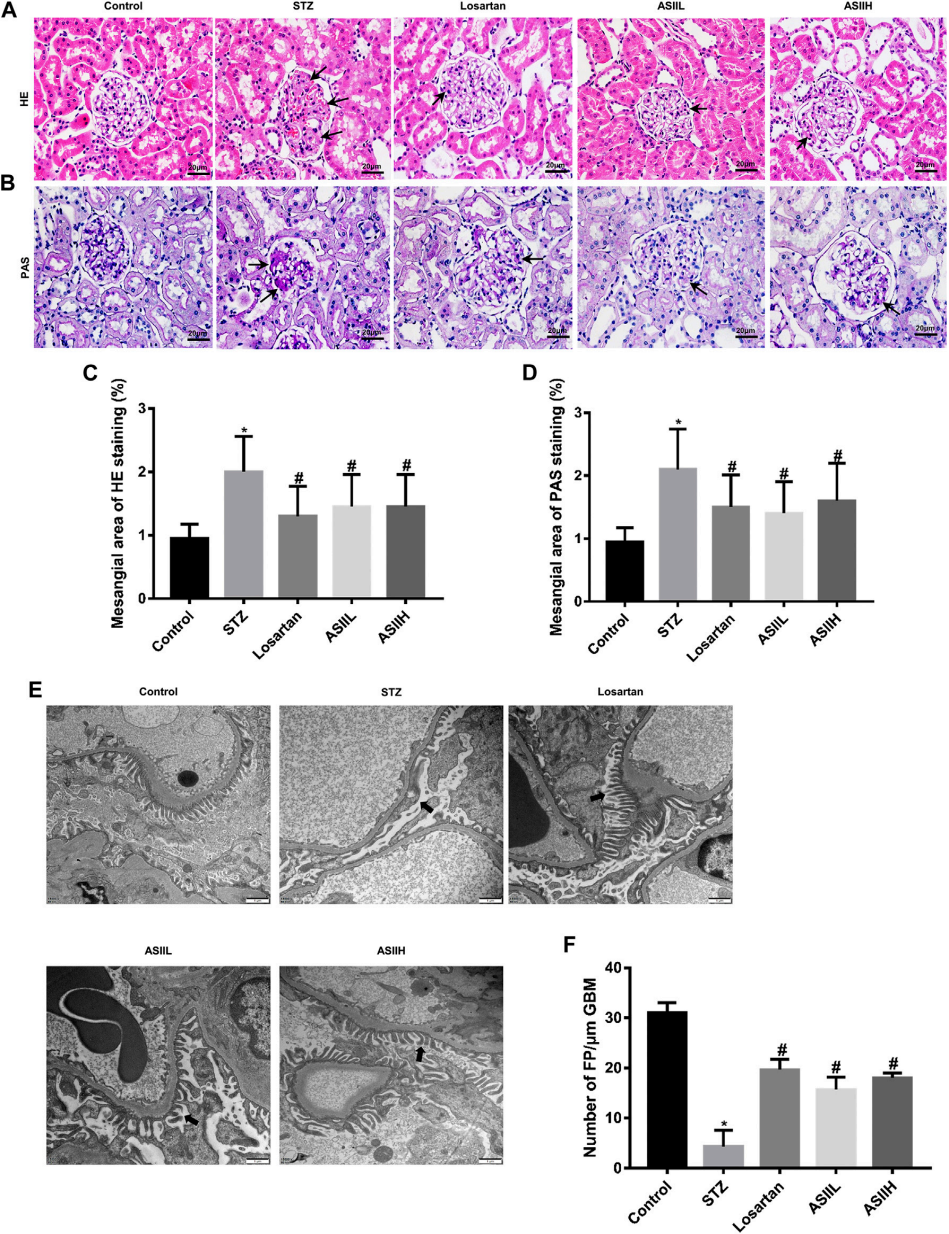
AS II alleviated renal histopathology and podocyte injury in STZ-induced diabetic rats. **(A, B)** Representative HE and PAS staining of glomerular and extracellular matrix expansion and accumulation (original magnification ×400). Black arrowheads indicated mesangial expansions. **(C, D)** Semiquantitative analysis of mesangial area changes in HE and PAS staining, respectively. **(E, F)** Ultrastructure photos of glomerular podocytes taken by transmission electron microscopy (TEM) (original magnification ×7,000, bars = 1 µm) and its semiquantitative analysis of podocyte FP density. Black arrowheads indicated changes in podocyte morphology. All data were expressed as mean ± SD. ∗*p* < 0.05 vs. control group; #*p* < 0.05 vs. STZ group.

We performed histological examination of renal tissues from all groups. As shown in HE and PAS staining (Figures 2A,B), diabetic rats were characterized with severe pathological changes, such as the accumulation of extracellular matrix (ECM) deposition, mesangial matrix expansion, and cell proliferation. For AS II- and losartan-treated diabetic rats, the situation of these pathological changes was significantly improved, as shown by quantitative analysis (Figures 2C,D). Furthermore, we observed the changes in podocytes morphology through TEM to further verify the role of AS II in DN progression. As presented in Figures 2E,F, the diabetic rats exhibited apparent podocyte loss, FP fusion, and effacement at 9 weeks after STZ injection. However, AS II treatment considerably reversed these changes in STZ-induced diabetic rats.

**FIGURE 3:**
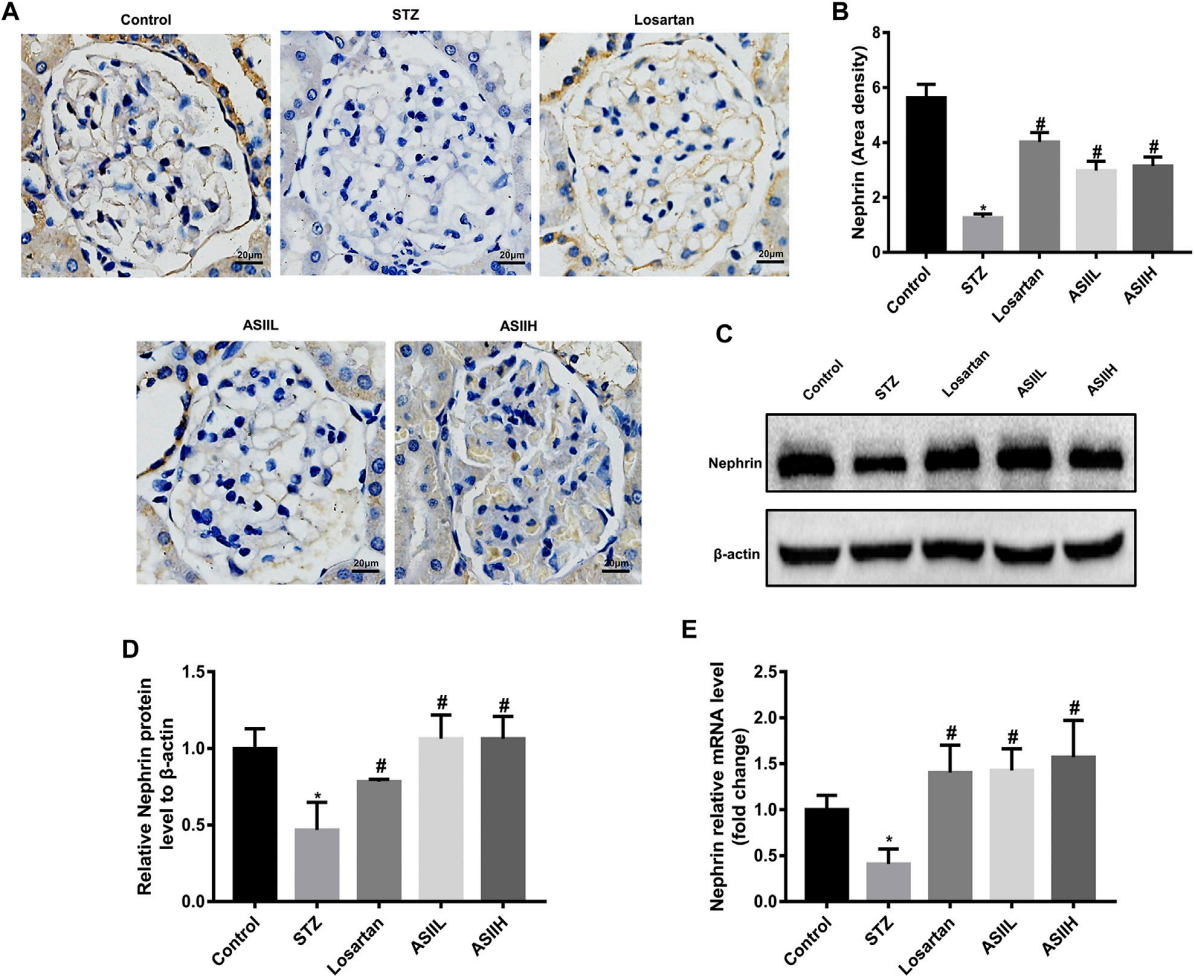
AS II restored nephrin protein expression in diabetic rats. **(A, B)** Representative photomicrographs of immunohistochemistry staining of nephrin (original magnification ×400) and the quantitative analysis of nephrin-positive area in each group. **(C, D)** The protein expression of nephrin was demonstrated by western blot and semiquantitative analyses in each group (fold change); β-actin served as a loading control. **(E)** Quantitative analysis of the mRNA level of nephrin in different groups. All data were expressed as mean ± SD. ∗*p* < 0.05 vs. control group, #*p* < 0.05 vs. STZ group.

To further assess the protective effects of AS II on podocytes in diabetic rats, we analyzed the expression of nephrin, which is a podocyte-specific protein and essential for the functional and morphological integrity of glomerular filtration barrier (Wang et al., 2018). As shown in Figures 3A,B, the number of nephrin-positive puncta was consistently decreased in diabetic rats compared with the normal rats, indicating the number of podocytes per glomerulus was dramatically downregulated and glomerular filtration barrier function was damaged. However, this change was notably revered by AS II and losartan treatment. Consistent with these observations, western blot and RT-qPCR results also confirmed that nephrin expression was decreased in diabetic rats, while AS II and losartan treatment remarkably reversed these changes (Figures 3C–E). These data suggested that AS II protected against diabetic podocyte abnormalities in function and structure.

### AS II Attenuated Podocyte Apoptosis in the Kidneys of Diabetic Rats

**FIGURE 4:**
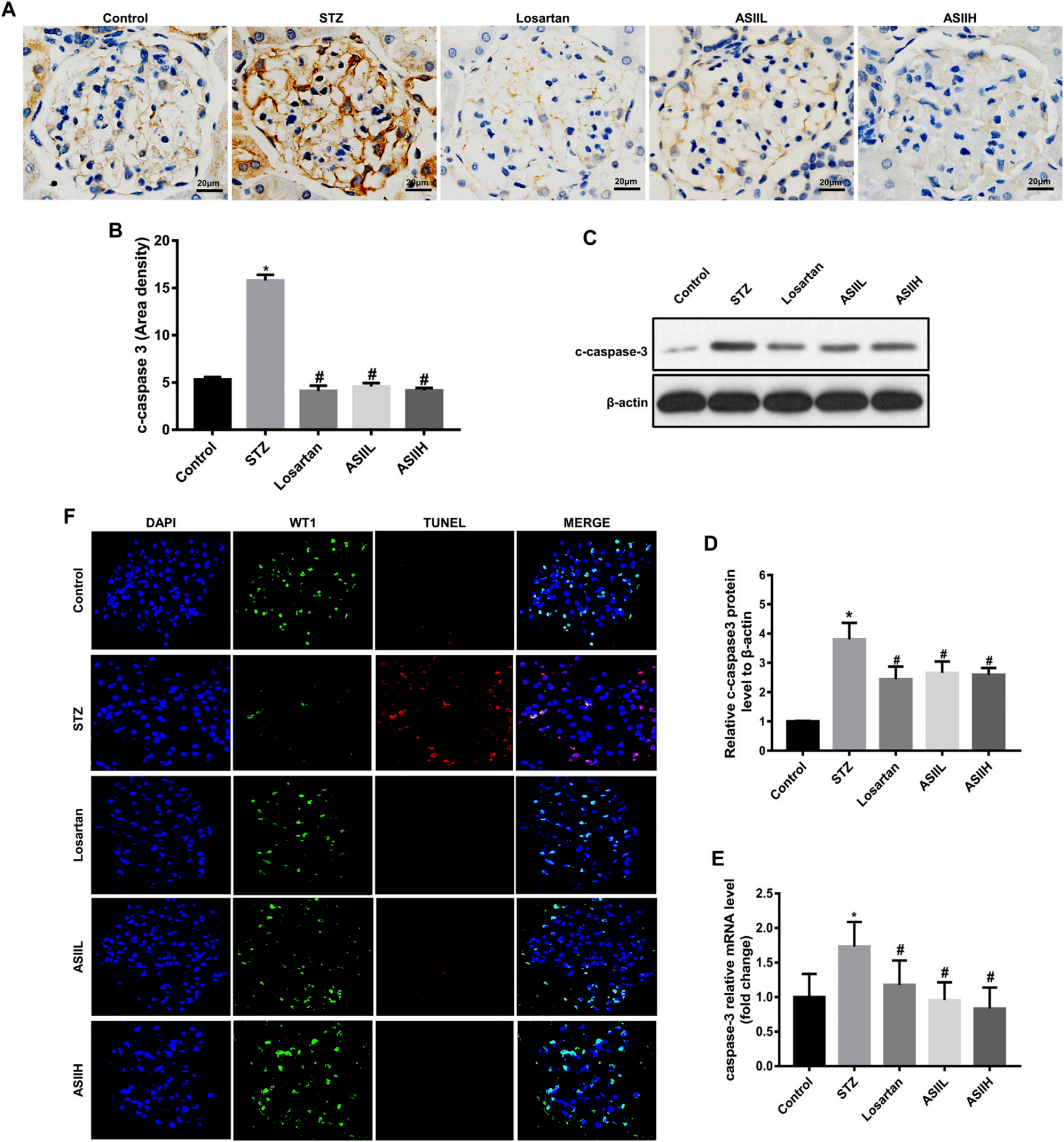
AS II attenuated podocyte apoptosis in diabetic rats. **(A, B)** Representative photomicrographs and semiquantitative analysis of c-caspase-3 by immunohistochemistry staining in each group (original magnification ×400). **(C, D)** The protein expression of c-caspase-3 was demonstrated by western blot and semiquantitative analyses in each group (fold change); β-actin served as a loading control. **(E)** Quantitative analysis of the mRNA levels of c-caspase-3 in each group. **(F)** Representative triple immunofluorescence labeling, including the TUNEL assay, WT1, and DAPI on frozen kidney sections. The cells with DAPI (blue), WT1 (green), and TUNEL (red) were identified as the apoptotic podocytes. Confocal microscopy was used to assess the rate of podocyte apoptosis: original magnification ×400, bars = 20 µm. All data were expressed as mean ± SD. ∗*p* < 0.05 vs. control group; ^#^ *p* < 0.05 vs. STZ group.

Triple immunofluorescence labeling, including TUNE, WT1, and DAPI, was used to examine the podocyte apoptosis in diabetic rats. Cleaved-caspase-3 (c-caspase-3) is an active form of caspase-3. It is well known for its role in apoptosis pathway, which is related to the deterioration of DN (Ghosh et al., 2009). In this study, we observed the expression of c-caspase-3 protein was significantly elevated in STZ-induced type 1 DN model compared to the normal rats, whereas AS II and losartan treatment dramatically reversed this change, as reflected by immunohistochemistry staining (Figures 4A,B). Moreover, western blot and RT-qPCR also showed the expression of c-caspase-3 was increased in diabetic rats, while AS II and losartan administration inhibited these effects (Figures 4C–E). Furthermore, double immunofluorescent staining of WT1 and TUNEL showed increased podocyte apoptosis in the kidneys of diabetic rats, but this change was reversed by AS II and losartan treatment (Figure 4F). These results demonstrated the significant antiapoptotic effect of AS II on podocytes in diabetic rats.

### AS II Restored Mitochondrial Morphology Changes and Dynamics-Associated Proteins Expression

**FIGURE 5:**
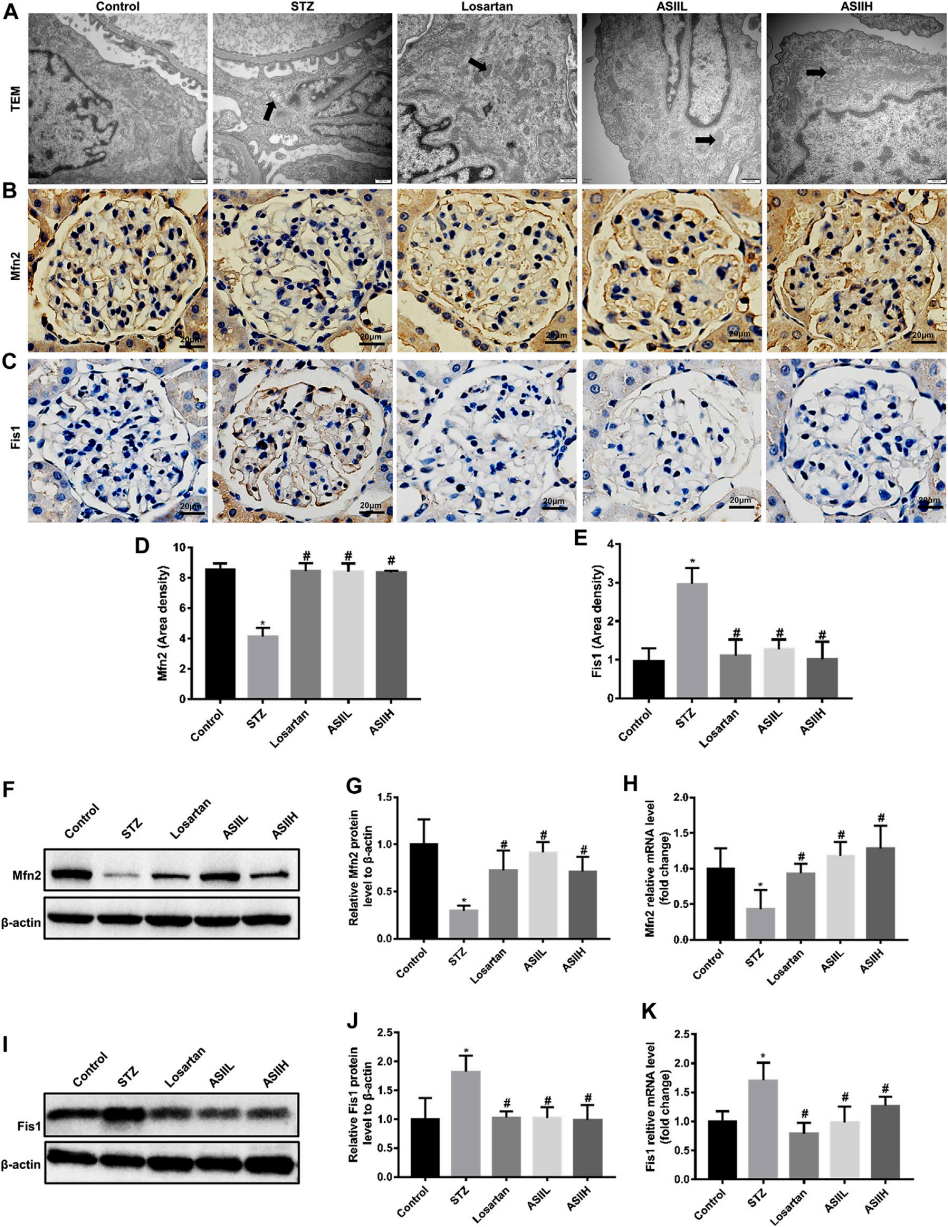
AS II restored the mitochondrial morphology and dynamics-associated proteins expression in diabetic rats. **(A)** Representative TEM micrographs of mitochondria morphology alterations in podocytes of each group (original magnification ×10,000, bars = 500 nm). **(B–E)** Representative photomicrographs and semiquantitative analysis of immunohistochemistry staining of Mfn2 and Fis1 (original magnification ×400). **(F–K)** Representative western blot and RT-qPCR analyses of Mfn2 and Fis1 in different groups (fold change); β-actin served as a loading control. Black arrowheads indicated changes in mitochondrial morphology. All data were expressed as mean ± SD. ∗*p* < 0.05 vs. control group; #*p* < 0.05 vs. STZ group.

To preferably understand the protective effects of AS II on mitochondrial biology, the changes of mitochondrial morphology and dynamics were observed by TEM, immunohistochemistry staining, western blot, and RT-qPCR assays. In Figure 5A, enlarged mitochondria in podocytes in parallel with the destruction of mitochondria cristae were found in diabetic rats, whereas these changes achieved amelioration partly after 9 weeks AS II and losartan treatment.

Given that Fis1 is essential for mitochondrial fission, while mitochondrial fusion is mediated by Mfn2, we then investigated the expression of Fis1 and Mfn2 to evaluate the effect of AS II on mitochondrial dynamics. The results showed that diabetic rats exhibited a significant reduction of Mfn2 protein, while Fis1 expression was upregulated compared with nondiabetic rats. However, AS II and losartan treatment increased the expression of Mfn2 and conversely decreased the expression of Fis1 in the kidneys of diabetic rats (Figures 5B–K). In summary, these data indicated the protective effect of AS II on mitochondrial morphology and dynamics changes in diabetic rats.

### AS II Ameliorated Autophagy Insufficiency and Restored Mitophagy-Associated Protein Expression in Diabetic Rats

**FIGURE 6:**
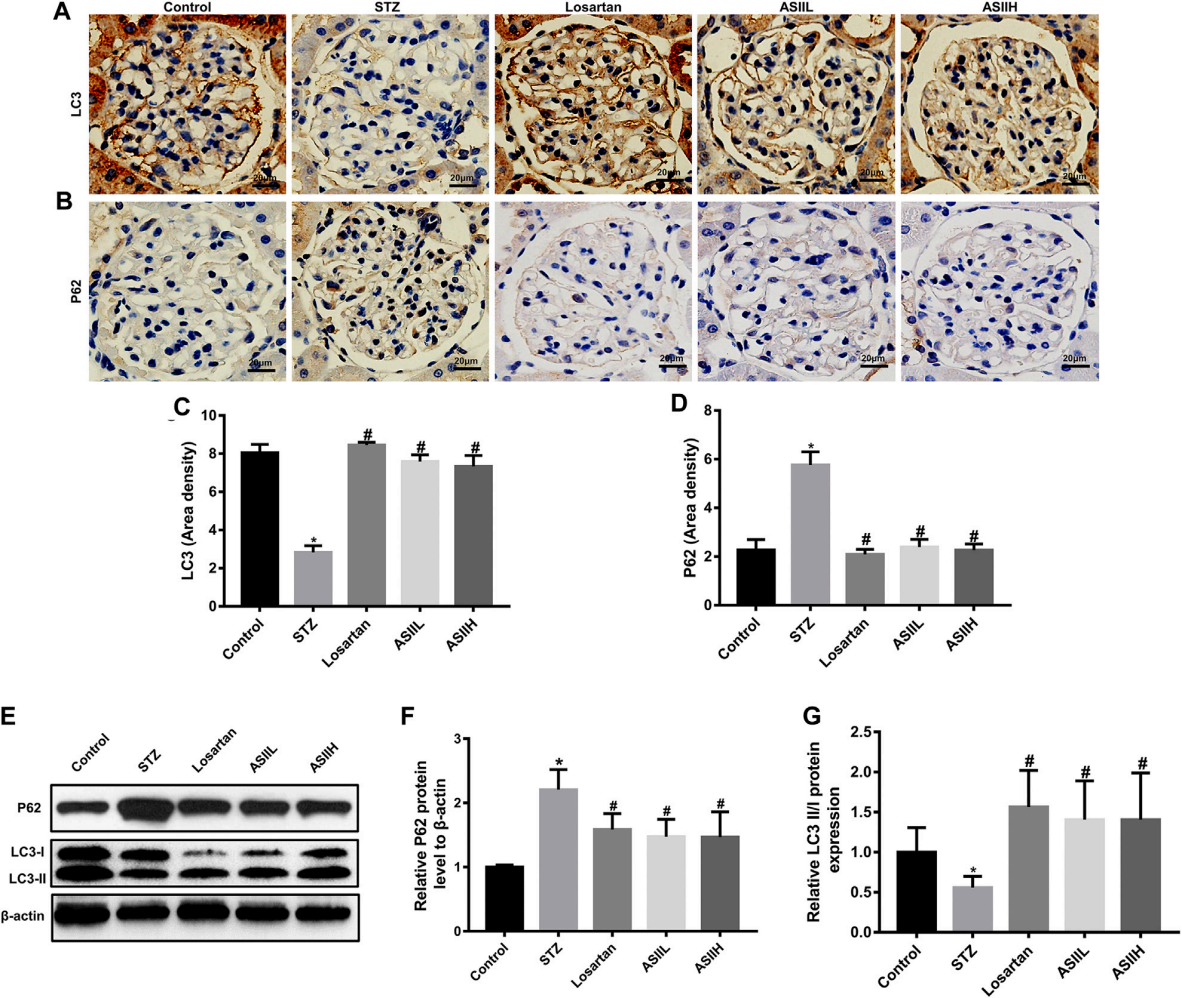
AS II ameliorated autophagy insufficiency through regulating autophagy-related protein expression. **(A–D)** Representative photomicrographs and semiquantitative analysis of immunohistochemistry staining for LC3 and P62 (original magnification ×400). **(E–G)** Western blot and semiquantitative analyses of LC3II/I ratio and P62 in each group (fold change). β-Actin served as a loading control. All data were expressed as mean ± SD. ∗*p* < 0.05 vs. control group; ^#^ *p* < 0.05 vs. STZ group.

We further examined the level of autophagy-related proteins in kidney tissues, such as LC3 and P62 proteins, to verify the effect of AS II on autophagy in DN. As is shown in Figures 6A–D, the expression of LC3 in diabetic rats was less than that in normal control rats. On the contrary, P62 expression was significantly increased after STZ injection. Nevertheless, AS II and losartan treatment obviously reversed the expression of LC3 and attenuated the level of P62 protein. Consistently, western blot results also showed that the ratio of LC3-II/LC3-I was decreased in diabetic rats, along with the increased P62 expression, while these alterations were partially reversed after 9-week treatment of AS II and losartan (Figures 6E–G). These data suggested that AS II exerted a beneficial effect on DN via reversing insufficient autophagy in diabetic rats.

**FIGURE 7:**
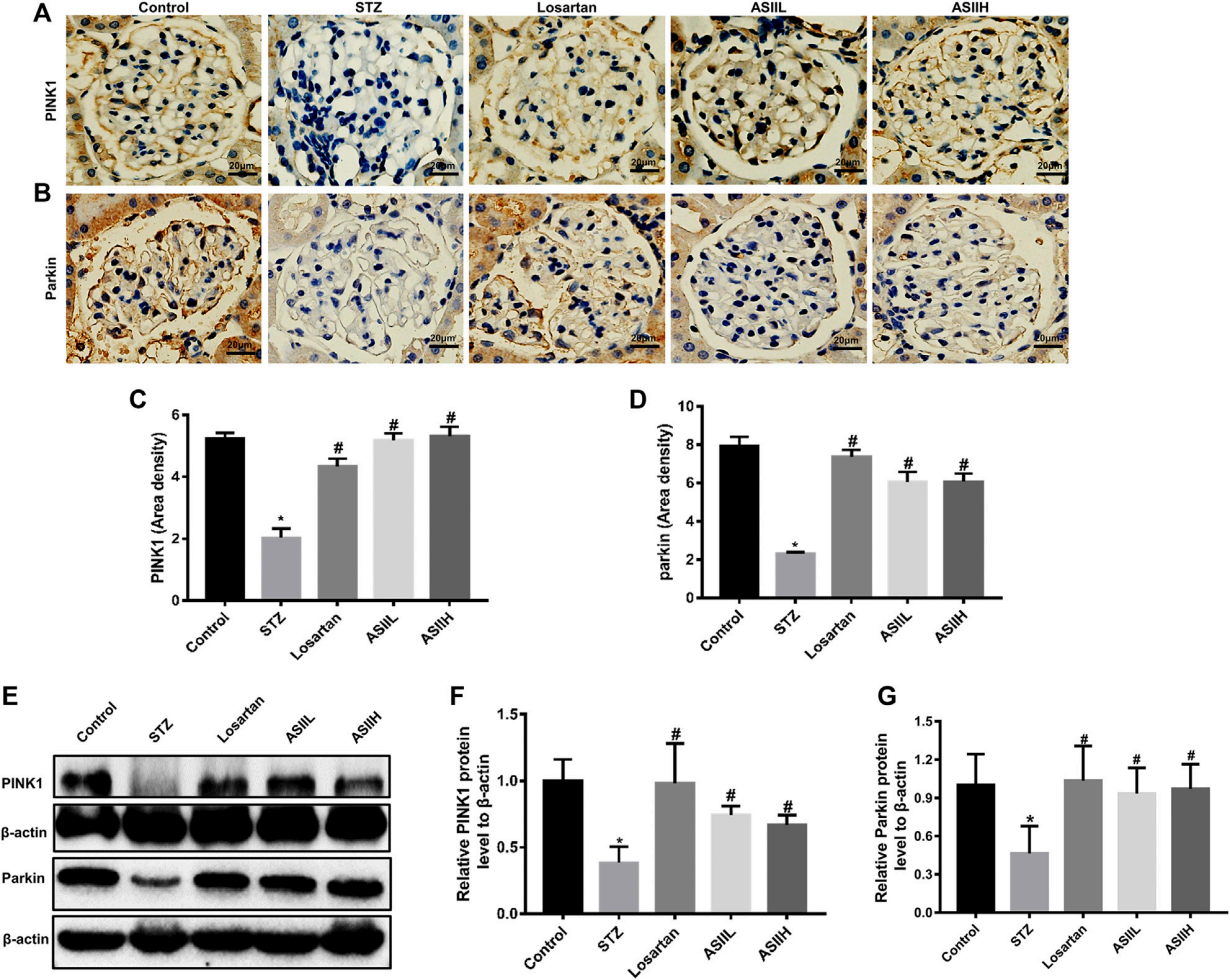
AS II stimulated the activation of mitophagy in diabetic rodents. **(A–D)** Representative photomicrographs and semiquantitative analysis of immunohistochemistry staining for PINK1 and Parkin (original magnification ×400). **(E–G)** Representative image of Western blot and semiquantitative analyses of PINK1 and Parkin in kidneys (fold change); β-actin served as a loading control. All data were expressed as mean ± SD. ^∗^ *p* < 0.05 vs. control group; ^#^ *p* < 0.05 vs. STZ group.

**FIGURE 8:**
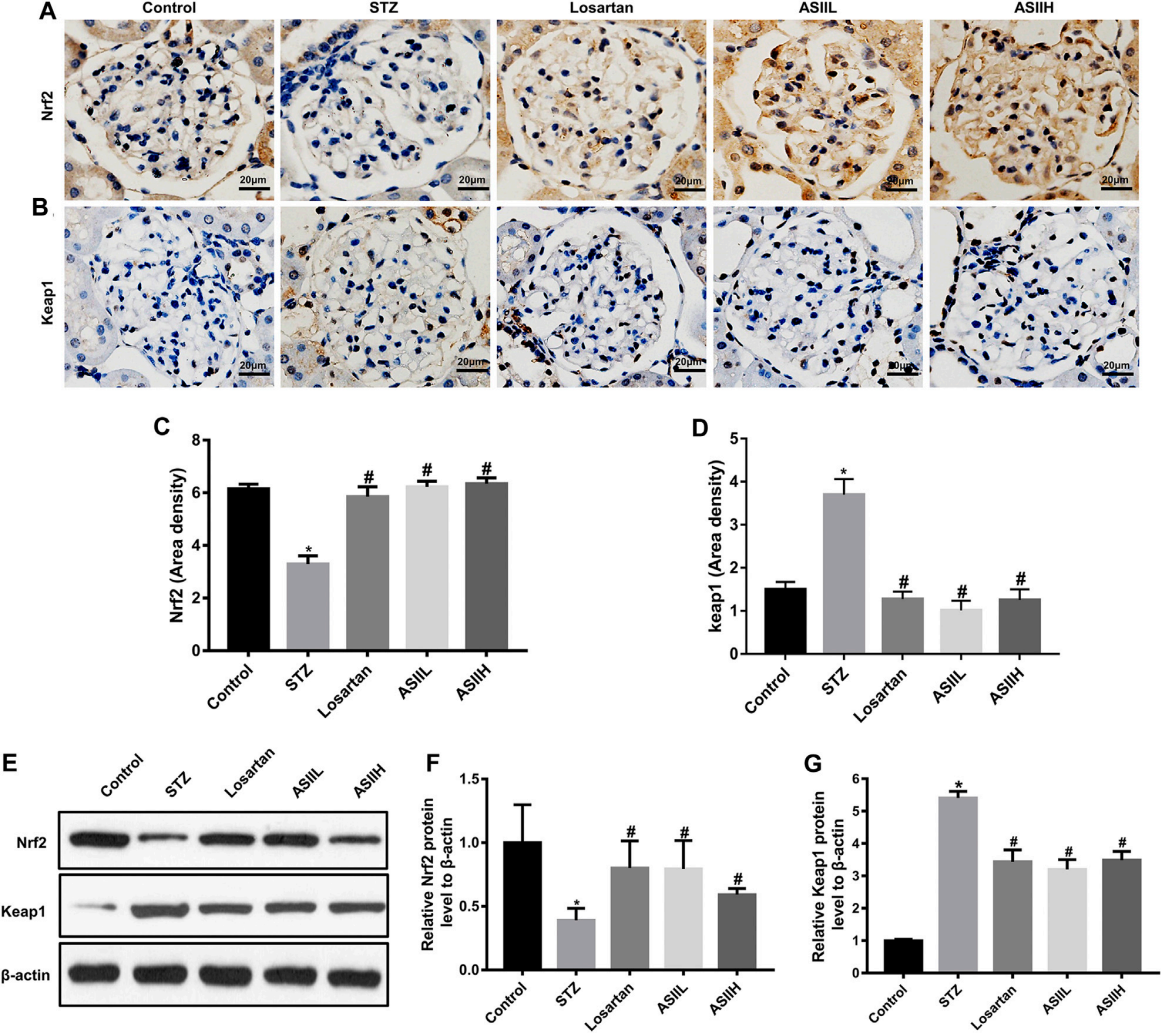
Effects of AS II on oxidative stress in diabetic rats. **(A–D)** Representative photomicrographs and semiquantitative analysis of immunohistochemistry staining for Nrf2 and Keap1 (original magnification ×400). **(E–G)** Western blot and semiquantitative analyses of Nrf2 and Keap1 in each group (fold change); β-actin served as a loading control. All data were expressed as mean ± SD. ∗*p* < 0.05 vs. control group; ^#^ *p* < 0.05 vs. STZ group.

Moreover, the expressions of PINK1 and Parkin related to mitophagy were apparently decreased in diabetic rats, whereas AS II and losartan treatment led to the enhancement of PINK1 and Parkin expression (Figures 7A–G). Because the expression of PINK1 is positively regulated by Nrf2 under oxidative stress conditions (Liu et al., 2018), we then investigated the level of Nrf2 and its upstream regulator Keap1 in the kidneys of diabetic rats. In our study, both immunohistochemistry staining and western blot results showed that the expression of Nrf2 was downregulated in diabetic rats, accompanied by increasing Keap1 level. However, AS II and losartan treatment increased the expression of Nrf2, further decreasing Keap1 level (Figures 8A–G). Taken together, the above results elucidated that the enhancement of antioxidant stress ability and mitophagy activation through comodulation of Nrf2 and PINK1 might be one of the possible mechanisms for the renoprotective effects of AS II.

## Discussion

Astragalosides have a wide range of pharmacological properties, such as anti-inflammatory, antiviral, and immunomodulatory activities (Lee et al., 2017; Qi et al., 2017). Astragaloside IV, one of the saponins from *Astragalus membranaceus*, has been reported to protect against DN in animal models (Wang et al., 2015; Song et al., 2018; Fan et al., 2019). However, the beneficial effects of AS II on podocyte injury and mitochondrial dysfunction in DN have not been studied yet. In this study, we investigated the protective effect of AS II on mitochondrial dynamics-related and autophagy-related proteins, as well as podocyte apoptosis and morphological changes in STZ-induced diabetic rats. The main findings of our study were that AS II ameliorated podocyte injury and mitochondrial dysfunction in diabetic rats partly through regulation of Nrf2 and PINK1 pathways. These novel findings might provide a promising therapeutic approach for the treatment of DN.

Firstly, we examined the protective effect of AS II on albuminuria in diabetic rats. According to the recent KDIGO 2020 Clinical Practice Guideline (2020), ACEi or angiotensin receptor blocker (ARB) therapy is recommended for patients with diabetes and chronic kidney disease. As one of the ARBs, losartan has been reported to have significant renal benefits in both type 1 and type 2 diabetic patients with nephropathy (Andersen et al., 2000; Brenner et al., 2001). Moreover, losartan was used as a positive control in our previous study (Zhai et al., 2019). Thus, we used losartan as a standard in this study. AS II significantly decreased the ratio of urine albumin to urine creatinine (ACR) in diabetic rats at 9 weeks after STZ injection. AS II also ameliorated glomeruli pathological changes. These results demonstrated that AS II delayed the progression of DN through improving renal abnormalities in function and structure in diabetic rats.

Secondly, we investigated the beneficial effect of AS II on podocyte injury and mitochondrial dysfunction in diabetic rats. Podocyte apoptosis is considered to be the major mechanism of podocyte loss, which exacerbated renal function decline and proteinuria (Li et al., 2019). Podocyte apoptosis is determinant in DN progression (Lei et al., 2018). Cleaved-caspase-3 (c-caspase-3) protein is the most important apoptosis executing protease in the cascade of apoptosis (Gu et al., 2016). Thus, we detected the renal expression of c-caspase 3. Using Triple immunofluorescence labeling including TUNE, WT1, and DAPI in kidney tissues, we further confirmed that AS II inhibited podocyte apoptosis in diabetic rats. Taken together, AS II attenuated podocyte apoptosis in the kidneys of diabetic rats.

We next investigated the effects of AS II on autophagy in diabetic rats. Autophagy is a highly regulated lysosomal degradation system (Gong et al., 2019). It is a process of degrading and recycling superfluous or damaged organelles or proteins in cells (Gong et al., 2019). Dysregulation of autophagy might contribute to glomerular pathological injuries under diabetic conditions (Yang et al., 2018). The rate of materials degradation via autophagy is called autophagy flux, which is monitored by measuring LC3 and P62 expression to distinguish autophagosomes from autolysosomes (Lumkwana et al., 2017; Kim et al., 2018). Notably, the number of autophagosomes is positively correlated with the level of LC3-II (Lumkwana et al., 2017). Moreover, P62 is an autophagy receptor, which served as an indicator of autophagic degradation (Kim et al., 2018). Therefore, we evaluated the level of autophagy in kidney tissues via detecting the ratio of LC3-II/LC3-I and P62 expression. The disturbances in mitochondrial homeostasis play a critical role in the pathogenesis of DN (Higgins and Coughlan, 2014). In addition, mitophagy is a kind of selective autophagy that eliminates damaged or dysfunctional mitochondria; thus, impairment of the mitophagy system leads to the aggravated progression of DN (Higgins and Coughlan, 2014; Sharma, 2017). As a regulated factor of mitophagy, PINK1 promotes the selective removal of mitochondria via displaying outer membrane accumulation and initiating Parkin protein translocation (Ren et al., 2017). In our study, decreased expression of PINK1 and Parkin protein in diabetic rats predicted the impairment of mitophagy, which was ameliorated by AS II treatment. Furthermore, mitophagy deficiency leads to damaged mitochondria accumulation, resulting in oxidative stress in the kidney (Flemming et al., 2018). Xiao et al. (2017) indicated that Nrf2 protein positively regulated the expression of PINK1 under oxidative stress conditions. Nrf2 is a key factor associated with oxidative stress and regulated by Keap1 (Kim et al., 2018). Thus, it is likely that AS II exerts beneficial effects on mitophagy through enhancing the ability of antioxidative stress via modulation of Nrf2 and PINK1 pathways.

There is mounting evidence of gender-specific aspects in renal diseases. It was reported that intact males developed proteinuria and kidney injury, whereas females were protected from injury (Baylis, 1994). Both our previous study (Zhai et al., 2019) and the other study (Thomson et al., 2016) used male rats to establish STZ-induced diabetic models. Therefore, only male rats were used in our study, and severe hyperglycemia and albuminuria and increased podocyte apoptosis were developed in male diabetic rats. By referring to published literature (Lee et al., 2017), we used different doses of AS II in our preliminary experiments. We found that 3.2 and 6.4 mg/kg of AS II could ameliorate albuminuria and did not cause apparent toxicity to the kidney. Therefore, we selected these two doses of AS II in this study.

Our previous study found that *Astragalus membranaceus* ameliorated albuminuria in diabetic rats and did not cause apparent toxicity to the kidney and liver (Zhai et al., 2019). Our clinical study further demonstrated that aqueous extract of Astragali Radix (ARE), the root of *Astragalus membranaceus*, induced obvious natriuresis and did not markedly affect mean arterial blood pressure, heart rate, and plasma concentration of creatinine in healthy men, suggesting that ARE is a safe natriuretic agent (Ai et al., 2008). A previous study reported that 30 or 50 mg/kg of AS II had very limited system toxicity on mice (Qiao et al., 2019). In this study, only 3.2 or 6.4 mg/kg of AS II was administered to the diabetic rats and these doses of AS II did not cause apparent toxicity to the kidney as shown in HE and PAS staining about the injury of renal histology. Thus, these findings suggested that AS II might be safe for the treatment of DN.

However, there are limitations in our study. Firstly, we only investigated the effects of AS II *in vivo*, and additional studies are needed to determine the underlying mechanism of AS II *in vitro*. Secondly, we did not investigate the effects of AS II on the normal control group and these effects will be investigated in further study.

Taken together, this study clearly demonstrated that AS II ameliorating podocyte injury and mitochondrial dysfunction in STZ-induced diabetic rats partly through comodulation of Nrf2 and PINK1. These novel findings might pave the way to a novel therapeutic strategy for the treatment of DN.

## Data Availability

The raw data supporting the conclusions of this article will be made available by the authors without undue reservation.
